# *McWRKY43* Confers Cold Stress Tolerance in *Michelia crassipes* via Regulation of Flavonoid Biosynthesis

**DOI:** 10.3390/ijms25189843

**Published:** 2024-09-12

**Authors:** Qiuxiu Yu, Caixian Liu, Jiahui Sun, Minghai Ding, Yu Ding, Yun Xu, Jinsong He, Qizhen Li, Xiaoling Jin

**Affiliations:** 1 College of Landscape Architecture, Central South University of Forestry and Technology, Changsha 410004, China; yqx25154x@163.com (Q.Y.); liucaixian2008@126.com (C.L.); 15966935601@163.com (J.S.); dingmh0321@163.com (M.D.); forgdfgual@gmail.com (Y.D.); xuyunandxuyun@163.com (Y.X.); hjs1802893476@126.com (J.H.); 2 Hunan Big Data Engineering Technology Research Center of Natural Protected Areas Landscape Resources, Changsha 410004, China; 3 Yuelushan Laboratory Carbon Sinks Forests Variety Innovation Center, Changsha 410004, China

**Keywords:** WRKY, cold stress, flavonoid biosynthesis, *McLDOX*, *Michelia crassipes*

## Abstract

WRKY transcription factor (TF) plays a crucial role in plant abiotic stress response, but it is rarely reported in *Michelia crassipes*. Our studies have found that the transcription factor *McWRKY43*, a member of the IIc subgroup, is strongly upregulated under cold stress. In this study, we cloned the full length of *McWRKY43* to further investigate the function of *McWRKY43* in resistance to cold stress and its possible regulatory pathways in *M. crassipes*. Under cold stress, the seed-germination rate of transgenic tobacco was significantly higher than that of the wild type, and the flavonoid content, antioxidant enzyme activities, and proline content of transgenic tobacco seedlings were significantly increased, which promoted the expression of flavonoid pathway structural genes. In addition, the transient transformation of *McWRKY43* in the *M. crassipes* leaves also found the accumulation of flavonoid content and the transcription level of flavonoid structural genes, especially *McLDOX*, were significantly increased under cold stress. Yeast one-hybrid (Y1H) assay showed that *McWRKY43* could bind to *McLDOX* promoter, and the transcription expression of *McLDOX* was promoted by *McWRKY43* during cold stress treatment. Overall, our results indicated that *McWRKY43* is involved in flavonoid biosynthetic pathway to regulate cold stress tolerance of *M. crassipes*, providing a basis for molecular mechanism of stress resistance in *Michelia*.

## 1. Introduction

With the frequent occurrence of extreme climates, plants are exposed to various stresses of abiotic environmental conditions, such as cold, drought, and salinity, which severely limit plant growth, development, and ornamental value [1,2]. Cold stress is one of the main abiotic stresses that can directly inhibit the plant’s own metabolic reaction and lead to plant cell dehydration, wilt, and even death [3,4]. Therefore, it is urgent to study the resistance mechanism of plants to cold stress and enhance plants’ tolerance to extreme temperature stress. As key regulatory proteins, transcription factors, including WRKY, MYB, NAC, bZIP, and other gene families, can not only sense abiotic stress signals and mediate physiological processes by regulating gene transcription, but can also promote the biosynthesis of secondary metabolites such as flavonoids, lignin, etc., to further enhance plant stress resistance [5,6,7,8].

WRKY is a key plant-specific transcription factor (TF) named for its characteristic WRKY domain WRKYGOK [9]. WRKY TFs can be classified into three groups (I, II, and III) based on the number of WRKY domains and the type of zinc finger motif. WRKY TFs in most plants have more Group II members, and they are further subdivided into five subgroups based on phylogenetic relationships: IIa, IIb, IIc, IId, and IIe [10,11]. Among them, the IIc subgroup is widely involved in plant growth and adaptation to the environmental abiotic stress response process, as a positive or negative regulatory factor [12]. The overexpression of *VvWRKY43* can enhance the tolerance of *Arabidopsis thaliana* under cold stress [13], and *CsWRKY43* is a positive regulator of drought resistance in *Camelina sativa* [14]. Other members of the IIc subgroup, *GbWRKY20* and *CpWRKY71*, were also strongly expressed under cold stress [15,16]. Under stress conditions, plants can initiate various changes at the physiological levels in response, including stomatal closure, reduced photosynthesis, and increased osmotic pressure accumulation. Under cold stress, the effects of plum *PmWRKY57* [17] and mantree *KoWRKY40* [18] on improving plant cold tolerance were studied. The results showed that the MDA content and H_2_O_2_ content in transgenic *A. thaliana* were significantly lower than wild type (WT), while the proline content, SOD, POD, and CAT activities of transgenic *Arabidopsis* were higher. The *PeWRKY48* gene was highly upregulated under cold stress in passion fruit. When subjected to cold stress, WT plants withered, while transgenic plants were not damaged by freezing. Physiological index results showed that the cold resistance of overexpressed *PeWRKY48* plants was further improved. For example, the content of MDA in transgenic plants was lower, while the content of PRO was higher than WT [9]. Taken together, low temperature is a common abiotic stress factor throughout the life cycle of plants, and the changes of related physiological indexes can directly reflect the damage degree of plants under adverse growth conditions [19].

In addition to regulating various stress responses, WRKY TF is also one of the largest gene families of secondary metabolites such as phenols, flavonoids, lignin, and tannins [9]. In apples, *MdWRKY11*, *MdWRKY40,* and *MdWRKY72* can regulate anthocyanin accumulation. *MdWRKY75*, a WRKY group IIc TF, indirectly stimulated anthocyanin accumulation by binding to *MdMYB1* promoter [20]. As an important secondary metabolite, flavonoids play a variety of roles in plants. More and more attention has been paid to their role in protecting against biological and abiotic stresses. WRKY TFs in IIc subgroup promoted the accumulation of flavonoids by upregulating the expression of CHI and CHS mediated by *GhMYC2*, thereby regulating the resistance of cotton to *Fusarium oxysporum* [21]. In abiotic stress, low temperature is known to induce the biosynthesis of flavonoid compounds such as anthocyanins, and the combination of anthocyanins and flavonols helps to limit the overexcitation of chlorophyll under cold stress [22]. Metabolomic and transcriptomic co-expression analysis of two different passion fruit resistance genotypes revealed that *PeWRKY30* is a key TF co-expressed with flavonoid accumulation in passion fruit and may be involved in biological or abiotic resistance of passion fruit [9]. In response to low-temperature regulation, *FaWRKY71* induced anthocyanin accumulation by promoting the expression of structural genes *FaF3′H*, *FaLAR*, *FaANR,* and transport factors *FaTT19* and *FaTT12* in the flavonoid pathway. Additionally, it mediated resistance to reactive oxygen species by increasing the enzyme activities of SOD, POD, and CAT, while decreasing MDA content [23]. In summary, these results support that WRKY transcription factors play an important role in the resistance to cold stress by promoting the accumulation of flavonoids, thereby enhancing the adaptability of plants to low temperature.

*Michelia crassipes* is a rare purple flower with a strong fragrance and a high ornamental value in *Michelia* Linn. But the stable inheritance of its flower color is also influenced by temperature. *M. crassipes* has a long history of cultivation in China, mainly in the Southern region. It prefers a warm and humid climate, and it is challenging to grow in the open fields north of the Yangtze River during winter (https://www.iplant.cn/frps/vol, accessed on 30 August 2024). With the frequent occurrence of extreme low temperatures in the Southern region, cold has become one of the main stress factors affecting the planting of *M. crassipes*. At the same time, due to the interference of climate and human activities, the natural population of *M. crassipes* has gradually decreased, and it is identified as an “endangered” plant (http://www.iplant.cn/rep/protlist/4, accessed on 30 August 2024). Therefore, it is of great significance to study the transcriptional regulation of the *M. crassipes* gene family under environmental stress for introduction and domestication and population conservation. Based on the existing transcriptome data [24], we reported for the first time that a WRKY transcription factor gene *McWRKY43*, which function was unknown, was involved in cold stress response in *M. crassipes*. Our results showed that *McWRKY43* is localized in the nucleus as a member of the WRKY IIc subgroup. In *M. crassipes*, the physiological and biochemical responses under cold stress were investigated by using tobacco leaf disc transformation and the transient transformation system. It was found that the overexpression of *McWRKY43* lines had stronger cold tolerance with higher antioxidant enzymes activity, flavonoid content, and flavonoid-related structural genes expression. Yeast one-hybrid (Y1H) assay demonstrated that *McWRKY43* could bind to the *McLDOX* promoter, and *McLDOX* was regulated by *McWRKY43* transcription under cold stress. Our findings provide new evidence that *McWRKY43* plays an important role in the synergistic regulation of low-temperature responses and flavonoid accumulation, and also provide a basis for the response mechanism of *M. crassipes* to abiotic stress.

## 2. Results

### 2.1. Extraction and Sequence Analysis of McWRKY43

**Figure 1:**
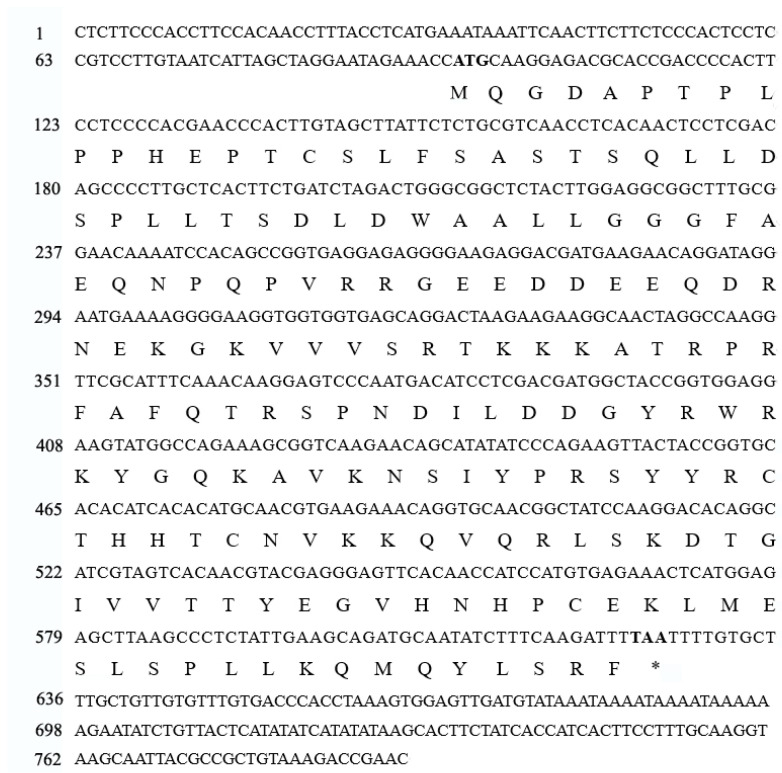
cDNA sequence of *McWRKY43* and its deduced amino acid sequence. The deduced amino acid sequence is shown underneath the corresponding nucleotide sequence. The others are noncoding regions, and the stop code was indicated with *.

Based on our previous transcriptome data of *M. crassipes*, which related to flavonoid biosynthesis during the color formation (SAR: PRJNA646857), the TF WRKY43 (Unigene7320), produced the significance difference under cold stress; we examined its full-length cDNA via RACE. In addition, the result illustrates that WRKY43 has an open reading frame (ORF) of 531 bp, a 5′ UTR (untranslated region) of 95 bp, and a 3′ UTR of 164 bp. Moreover, it encodes a 176 aa protein (Figure 1).

Using the web software ProtParam, we further revealed that *McWRKY43* has a molecular weight of 20,083.66 Da and a theoretical isoelectric point (pI) of 9.03. Based on this evidence, we identified this protein to be both unstable and hydropathic. Moreover, using the web softwares TMHMM server 2.0 and SignalP 5.0, we estimated that *McWRKY43* had no signal peptide and transmembrane sites. This suggests that *McWRKY43* is likely a nonsecretory and/or nontransmembrane protein.

**Figure 2:**
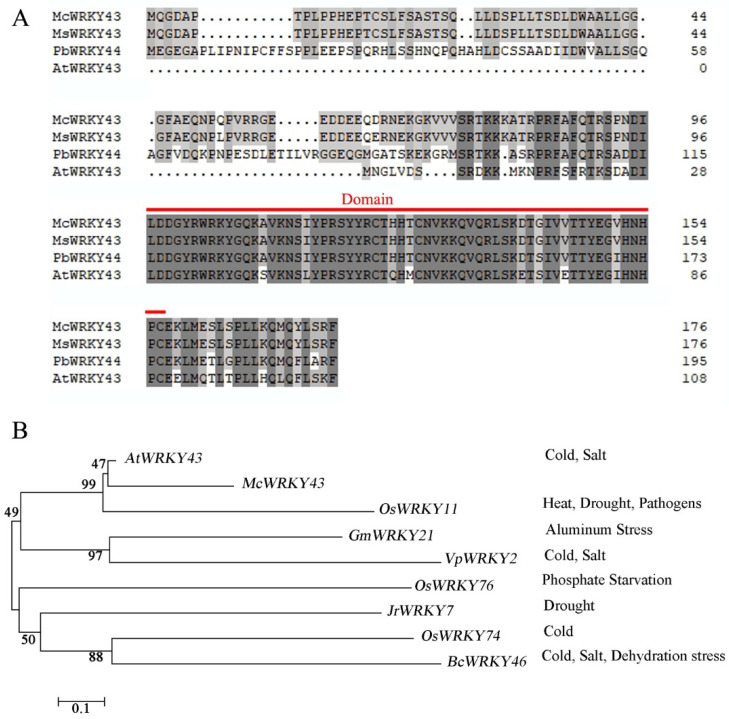
Characterization of *McWRKY43*. (**A**) Alignment of *McWRKY43* with *Magnolia sinica* WRKY43, *Phoebe bournei* WRKY44, and *Arabidopsis thaliana* WRKY43. Red line indicates the location of C-terminal WRKY DNA-binding domain, and identical amino acids are shaded in black. (**B**) Phylogenetic tree analysis of *McWRKY43* with other known stress-responsive WRKY proteins. Protein-accession numbers are as follows: *Arabidopsis thaliana* WRKY43 (AEC10647.1), *Oryza sativa* WRKY11 (DAA05076.1), *Glycine max* WRKY21 (ABC26913.1), *Vitis pseudoreticulata* WRKY2 (ADD70008.1), *Oryza sativa* WRKY76 (NP_001409692.1), *Juglans regia* WRKY7 (ALU11214.1), *Oryza sativa* WRKY74 (AAT84161.1), and *Brassica rapa* subsp. chinensis WRKY46 (ADM32893.1).

The homologous sequence alignments of *McWRKY43* with *Magnolia sinica* WRKY43 (XP_058096142.1), *Phoebe bournei* WRKY44 (URH10292.1), and *Arabidopsis thaliana* WRKY43 (AEC10647.1) were performed by DNAMAN5.2.2, and they shared a homology rate of 66.20%. Based on a sequence analysis, *McWRKY43* was shown to contain C-terminal WRKY DNA-binding domain, thereby suggesting that it is a WRKY TF (Figure 2A).

A phylogenetic tree based on the sequences of *McWRKY43* and some other known stress-related WRKYs was constructed, which revealed that *McWRKY43* was clustered closely to *Arabidopsis thaliana* WRKY43 (AEC10647.1) and *Oryza sativa* WRKY11 (DAA05076.1) (Figure 2B). The results suggest that *McWRKY43* may also be involved in the regulation of abiotic stress, especially cold and salt.

### 2.2. McWRKY43 Expression Pattern Analysis in Michelia crassipes Law

**Figure 3:**
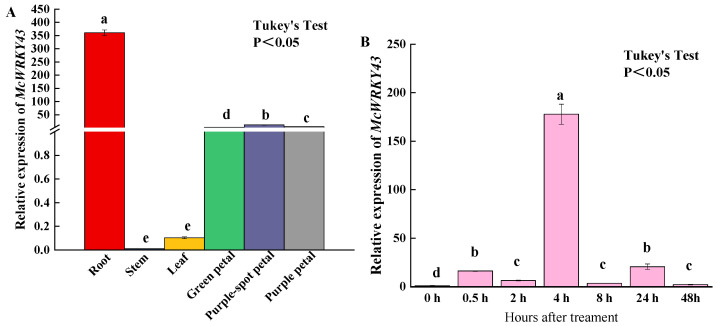
Expression characteristics analysis of *McWRKY43*. (**A**) Tissue-specific expression patterns of *McWRKY43*. (**B**) Relative expression levels of *McWRKY43* in *M. crassipes* under cold stress. The values represent the mean ± standard deviation (SD) of three biological replicates, and different letters above the bars as determined by Tukey’s test indicate significant differences at the *p* < 0.05 level.

To explore the expression patterns of *McWRKY43*, qRT-PCR was used to determine the transcript levels of *McWRKY43* in different tissues and perianth of different color stages in *M. crassipes*. The highest expression of *McWRKY43* was found in Root, followed by that in purple-spot tepal, purple tepal, green tepal, leaf, and stem (Figure 3A). Due to the regulation of *AtWRKY43* in cold, which was clustered closely to *McWRKY43*, we further examined the expression of *McWRKY43* during low temperature.

The *McWRKY43* expression pattern was determined in the *M. crassipes* leaves exposed to CS (Cold Stress) (Figure 3B). With increasing duration, the *McWRKY43* levels increased remarkably in cold stress, reaching their highest expression on the 4th hour post-exposure. Relative to the 0 h, the *McWRKY43* levels in *M. crassipes* exposed to CS were markedly high. Together, low temperature strongly promoted the expression of *McWRKY43*.

### 2.3. McWRKY43 Encodes a Nucleus-Localized Protein with Transcriptional Activation Activity

**Figure 4:**
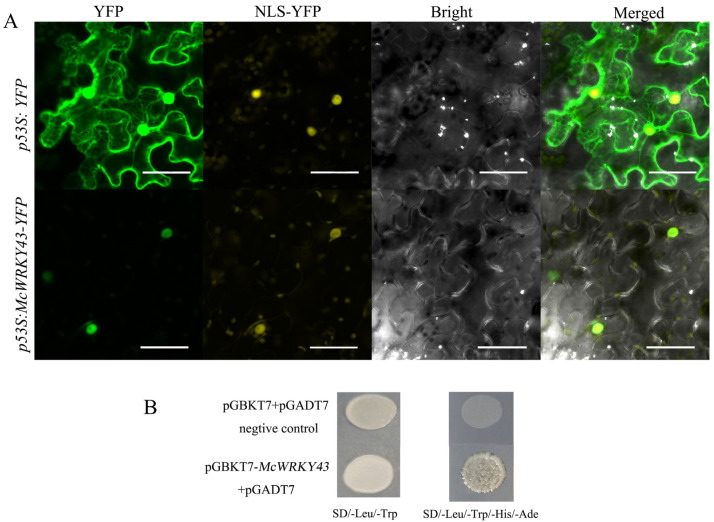
Nuclear localization and transactivation assay of *McWRKY43*. (**A**) Subcellular localization of *McWRKY43* in tobacco leaves. YFP, NLS-YFP, bright, and merged images were taken (Scale bar, 50 µm). YFP Control and YFP-MCWRKY43 were detected with green fluorescence signal, and NLS was detected with yellow fluorescence signal. (**B**) Transcriptional activation analysis of *McWRKY43* in yeast. Yeast strains that contained ‘empty’ pGADT7 and pGBKT7 plasmids were used as negative Controls.

The YFP-*McWRKY43* fusion construct and the YFP Control driven by CaMV35S promoter were transformed in tobacco epidermal cells, respectively, and visualized under a laser-scanning confocal microscope to determine the subcellular localization of *McWRKY43*. Results showed the fluorescence signal from YFP alone was widely distributed throughout the cells, whereas the YFP-*McWRKY43* fusion protein fluorescence signal was detected to coincide with NLS (Nuclear Localization Signal) (Figure 4A), which indicated that McWRKY43 is a nuclear protein.

By using the GAL4-based yeast system, we investigated the transcriptional activity of *McWRKY43* (Figure 4B). As expected, all the transformants grew well on the yeast synthetic drop-out medium lacking Leu and Trp (SD/-Leu/-Trp). However, only the yeast transformed with pGBKT7-*McWRKY43* grew well on the yeast synthetic drop-out medium lacking Leu, Trp, His, and Ade (SD/-Leu/-Trp/-His/-Ade), and the pGBKT7 vector barely grew at all on SD/-Leu/-Trp/-His/-Ade medium, suggesting *McWRKY43* had transcriptional autoactivation activity. 

### 2.4. McWRKY43 Positively Regulates Flavonoid Biosynthesis during Flowering under Cold Stress

**Figure 5:**
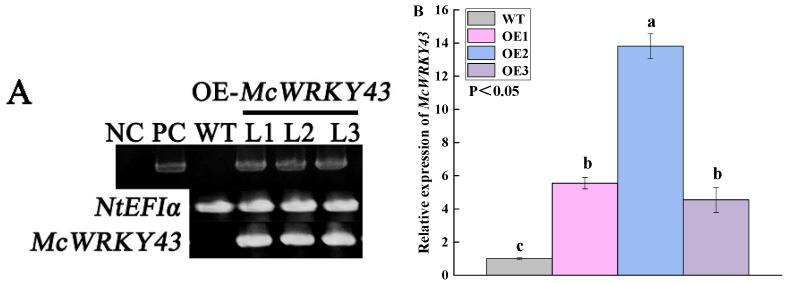
Identification of transgenic tobacco lines. (**A**) PCR analysis of *McWRKY43* DNA and mRNA. Note: From left to right are NC (negative Control: ‘empty’ pCAMBIA1300 plasmids), PC (positive Control: p35S-*McWRKY43* plasmid), WT (wild type), and OE-*McWRKY43*. (**B**) Relative expression level of *McWRKY43* in leaves by qRT-PCR; all data represent means ± SD of three biological replicates, and different letters indicate significant differences by Tukey’s test (*p* < 0.05).

PCR and qRT-PCR were used for the identification of transgenic tobacco plants in this study. The leaves of 2-month-old T1-generation transgenic tobacco were employed as materials for identification. The PCR analysis revealed that all the tobacco plants produced a single bright band of *NtEF1*α, and the products of *McWRKY43* were detected only in the transgenic lines (TL) but not in the wild type (WT) (Figure 5A). In addition, we performed a further analysis using qRT-PCR. We demonstrated that the *McWRKY43* levels in TL were markedly elevated relative to WT (Figure 5B). These results suggest that *McWRKY43* was successfully transformed in tobacco plants.

**Figure 6:**
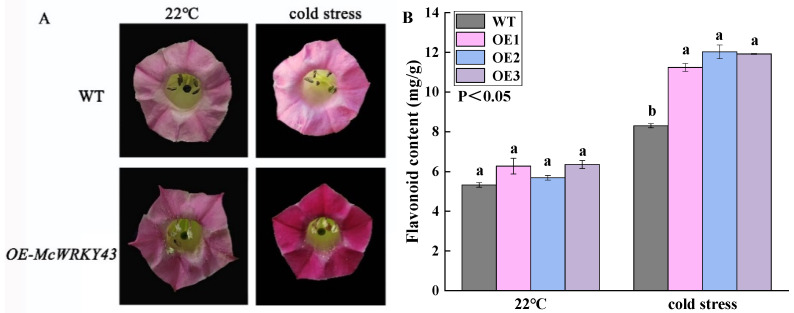
Changes of flower color and flavonoid content of wild type and transgenic tobacco under cold stress. (**A**) Flower color changes of wild type and transgenic tobacco before and after cold stress. (**B**) Changes of flavonoid content in the corolla of wild type and transgenic tobacco before and after cold stress. All data represent means ± SD of three biological replicates, and different lowercase letters indicate significant differences by Tukey’s test (*p* < 0.05).

The flavonoid contents of transgenic tobacco were slightly higher than wild type in a normal growth environment. However, under cold stress, it was found that the flower color of transgenic tobacco plants was significantly deepened (Figure 6A) and the flavonoid contents were increased significantly compared to the WT, with an increase of 41.23% (Figure 6B). These results indicated that *McWRKY43* transgenic tobacco may have a positive impact on the flavonoid biosynthesis pathway, especially in the low-temperature environment.

### 2.5. The Overexpression of McWRKY43-Enhanced Cold Stress in Tobacco

#### 2.5.1. Effect of Cold Stress on Seed Germination of *McWRKY43* Transgenic Tobacco

**Figure 7:**
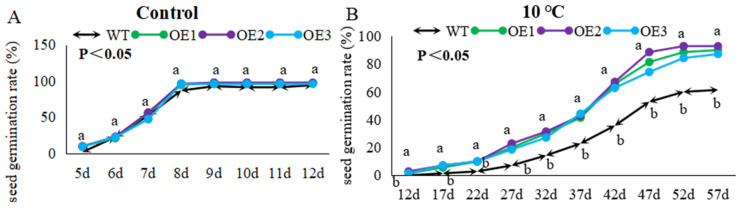
Comparison of seed-germination rate between WT and transgenic tobacco. (**A**) Germination rate of WT and transgenic tobacco seeds under a normal environment. (**B**) The germination rate of WT and transgenic tobacco at 10 °C. Values in (**A**,**B**) represent means of three replicates with 70 seeds per replicate, and different lowercase letters indicate significant differences by Tukey’s test (*p* < 0.05). In (**A**), both the WT and the OEs group are labeled as ‘a’. In (**B**), the letter ‘a’ indicates that the three transgenic lines have the same level of significance, and the letter ‘b’ indicates significant differences between the WT and all three OEs.

We tested the effect of cold on germination of *McWRKY43* overexpressed seeds. The results showed that there was no significant difference in the germination rate of WT and transgenic plants at 22 °C (Figure 7A). However, the germination rate of transgenic plants in the 10 °C low temperature was significantly higher than WT from the 12th day to the 57th day (Figure 7B, Table S6). After 52 days of cultivation, the seed-germination rate of each line tended to stabilize. The seed-germination rate of transgenic plants exceeded 84%, while the WT was only 60%. These results indicate that the overexpression of *McWRKY43* can significantly improve cold stress tolerance of tobacco plants.

#### 2.5.2. Physiological and Biochemical Analysis

**Figure 8:**
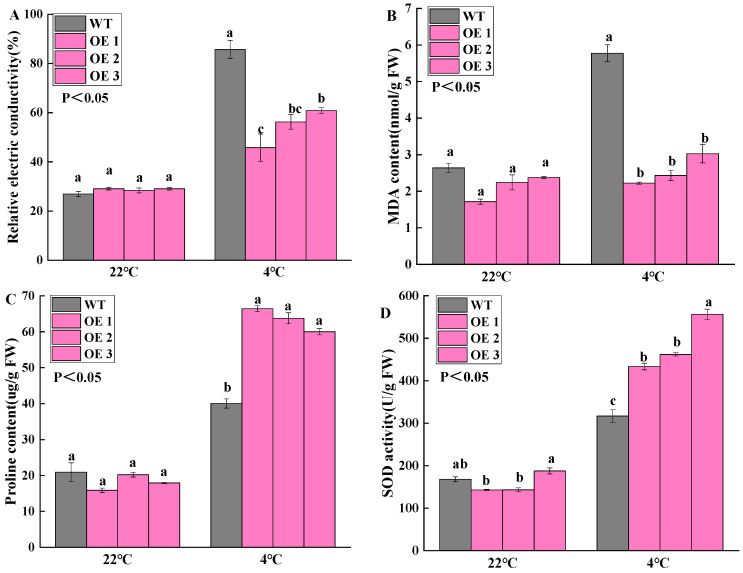

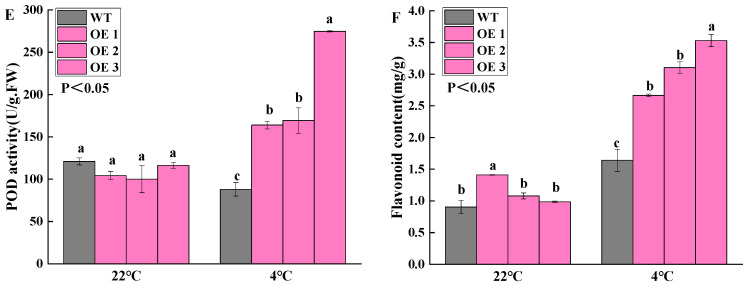
Measurement of physiological indices. (**A**–**F**) Relative electronic conductivity, MDA content, proline content, SOD activity, POD activity, and flavonoid content. The values represent the mean ± SD) of three biological replicates, and different letters above the bars as determined by Tukey’s test indicate significant differences at the *p* < 0.05 level at the same temperature.

Plants produce a large number of reactive oxygen species (ROS) under low-temperature stress, which changes the membrane permeability. To determine whether the *McWRKY43* gene can enhance plant resistance to cold, we examined the content of flavonoid, relative electric conductivity (REC), MDA, and proline content, as well as the activity of SOD and POD in both WT and overexpressed *McWRKY43* transgenic tobacco (OE) before and after exposure to cold stress. After 2 months of cultivation, the leaves of all the tobacco plants were used as materials. Next, the tobacco plants were exposed to cold stress at 4 °C for 24 h. Based on our results, the leaves of WT and OE showed massive differences. The WT leaves showed clear signs of wilting and drooping, whereas the OE grew normally (Figure S1). As shown in Figure 8, under normal growth conditions, there were no significant differences in REC, MDA, and free proline content, as well as the activities of SOD and POD in all tobacco samples. After exposure to cold stress, the transgenic tobacco showed significantly lower levels of REC and MDA compared to the WT, while the contents of free proline and flavonoids, SOD, and POD activities were significantly increased. These results showed that the *McWRKY43* gene reduced the damage of ROS to cell membrane permeability by increasing the activity of antioxidant enzymes, proline, and flavonoid content accumulation of transgenic tobacco, and thus improved the tolerance of tobacco to cold stress.

### 2.6. Altered Expression of Flavonoid Biosynthesis-Related Genes in McWRKY43 Transgenic Tobacco Plants under Cold Stress

**Figure 9:**
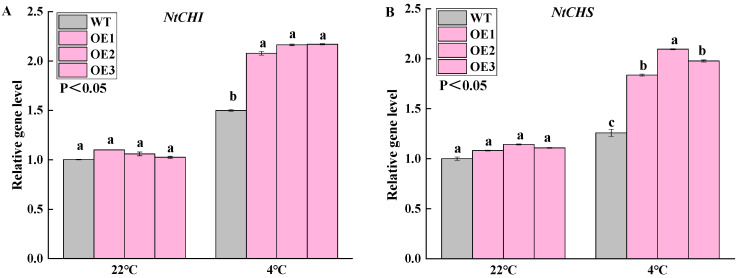

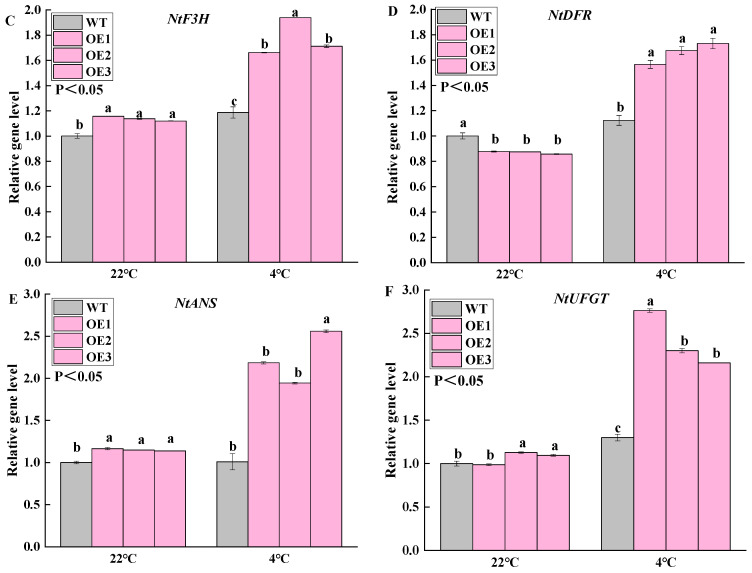
Transcript levels of the flavonoid biosynthesis-related genes under cold stress in WT and *McWRKY43* transgenic tobacco. (**A**–**F**) Transcript levels of *NtCHI, NtCHS*, *NtF3H*, *NtDFR*, *NtANS* and *NtUFGT*. The values represent the mean ± SD of three replicates, and different letters above the bars as determined by Tukey’s test indicate significant differences at the *p* < 0.05 level at the same temperature.

*McWRKY43* Transgenic tobacco plants showed improved cold tolerance, along with the accumulation of flavonoids. Therefore, transcription levels of a number of flavonoid biosynthesis-related genes were tested in cold-stressed tobacco, including *NtCHS*, *NtCHI*, *NtF3H*, *NtDFR*, *NtANS,* and *NtUFGT* genes. The qRT-PCR results showed higher transcripts of these genes accumulated in *McWRKY43* transgenic plants compared to WT in 4 °C (Figure 9). The expression levels of flavonoid biosynthesis-related genes in *McWRKY43* transgenic tobacco were similar to WT in 22 °C, but significantly higher than WT under cold stress. We speculated that *McWRKY43* might have flavonoid-mediated response to cold stress, and the activation and expression of these genes could potentially enhance the cold tolerance of transgenic plants.

### 2.7. McWRKY43 Conferred Cold Tolerance in M. crassipes

Transient transformation was used to confirm the function of the *McWRKY43* gene. The Agrobacterium-mediated transient expression system was used to deliver gene-expression vectors transiently to the abaxial side of leaves of *M. crassipes*. qRT-PCR was used for the identification of transgenic plants in this study. After 2 days, compared to the Controls, the *McWRKY43* levels were markedly high in the *M*. *crassipes* leaves infiltrated with the *McWRKY43* expression vector, with values that were, respectively, 14, 13, and 12 times (Figure S2E).

*M. crassipes* plants were exposed to cold stress for 24 h at 0 °C. MDA and electrolyte leakage index are important indicators for evaluating the membrane stability of plants under cold stress and their ability to resist low temperatures [25]. So, we evaluated the relative electric conductivity (REC) and MDA to assess the growth status of *M. crassipes* leaves post cold stress. As shown in Figure S3, there were no significant differences in two physiological indices at 22 °C. However, under cold stress, REC and MDA content in Control leaves were significantly higher than OE-*McWRKY43* (Figure S3A,B). The results showed that the cell membrane damage was reduced in the OE-*McWRKY43* groups under cold stress. At the same time, we found that transient overexpression of the *McWRKY43* gene increased flavonoid content, especially after cold stress, and the OE-*McWRKY43* group showed a very significant increase (Figure S3C).

### 2.8. The Overexpression of McWRKY43-Enhanced Expression of Flavonoid Biosynthesis-Related Genes in M. crassipes under Cold Stress

Many studies have shown that the flavonoid biosynthesis pathway can improve plant resistance by regulating the gene-transcription level. qRT-PCR analyses were performed for Control and OE-*McWRKY43* leaves at the different time in 0 °C. Several key structural genes involving flavonoid biosynthesis were selected for further analysis using standardized transcript levels, shown as heat maps (Figure S4A–C). These structural genes included *McPAL1*, *McPAL2*, *McPAL3*, *McPAL4*, *McPAL5*, *McCHS1*, *McC4H*, *McF3′H*, *McDFR,* and *McLDOX*. At 0 °C for 0.5 h, a group of EBG genes (*McPAL3*, *McPAL5*, *McCHS1,* and *McC4H*) and *McLDOX* showed significantly higher expression levels in OE-*McWRKY43* compared to Control. 

After 4 h of cold stress, the results showed higher transcripts of these genes, which included *McPAL1*, *McPAL2*, *McPAL4*, *McCHS1*, *McC4H,* and *McLDOX*, accumulated in *McWRKY43* transgenic leaves compared to Control. *McPAL4*, *McCHS1*, *McF3′H,* and *McLDOX* genes were still significantly expressed at 24 h. Furthermore, it was observed that both the *McCHS1* and *McLDOX* genes showed higher transcript levels in OE-*McWRKY43* during cold treatment. This indicates that *McWRKY43* may have the potential to enhance plants’ resistance to cold by binding to *McPAL2* and *McLDOX* to regulate to flavonoid biosynthesis pathways.

### 2.9. McWRKY43 Can Bind to the Promoter of McLDOX to Co-Regulate Cold Stress

**Figure 10:**
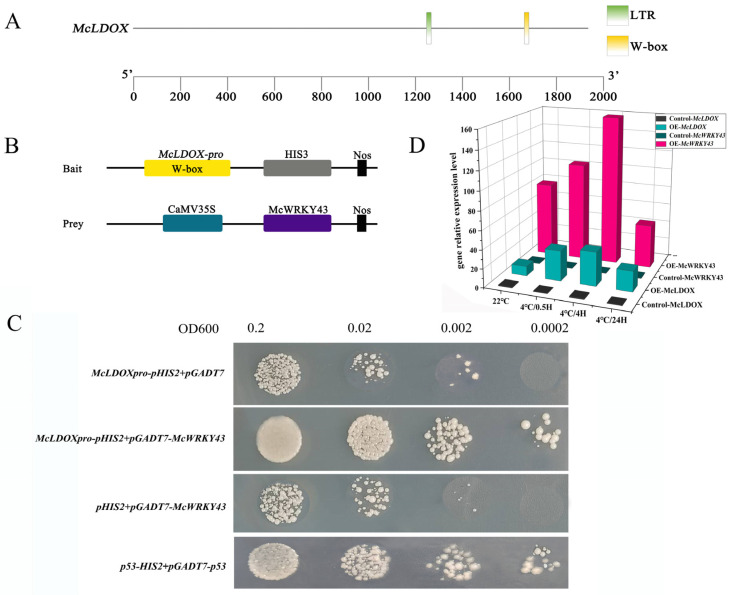
*McWRKY43* binds to the promoter of *McLDOX* and increases its expression. (**A**) Predicted cis elements on the *McLDOX* promoter. The bold black line and the colorful icons represent the promoter and different cis elements, respectively. (**B**)The pBait-HIS2 and pGADT7 prey vectors are shown. (**C**) *McWRKY43* binds to the W-box cis-acting element of the *McLDOX* promoter at different dilutions of SD/-Leu-Trp-His added with 50 mmol/L 3-AT. (**D**) The expression levels of *McWRKY43* and *McLDOX* genes changed during cold stress.

Herein, we amplified the sequence of promoter of *McLDOX* via Tail-PCR. Analysis by Newplace software (http://www.dna.affrc.go.jp/PLACE/, accessed on 20 July 2024) showed that the *McLDOX* promoter sequence contained WRKY recognition site (Figure S5) and one cis-acting element in response to low temperature (LTR) (Figure 10A). 

Then, we utilized the yeast one-hybrid (Y1H) assay to further confirm whether McWRKY43 regulates the transcription of *McLDOX* by binding to the W-box (TTGACC) in the *McLDOX* promoter. The minimal inhibitory concentration of 3-Amino-1,2,4-triazole (3-AT) for bait yeast strains was found to be 50 mmol/L. The pGADT7-*McWRKY43* and empty pGADT7 were transformed to the yeast strain Y187 carrying *McLDOX*-pro-pHIS2-Wbox plasmid, respectively, and it was found that the yeast strain could grow normally on the SD/-Leu-Trp medium. Only the yeast strain transformed with pGADT7-*McWRKY43*, in addition to the positive Control P53, was able to grow normally on the SD/-Leu-Trp-His medium with 50 mM 3-AT (Figure 10C).

At the same time, the results revealed that *McLDOX* showed high transcriptional expression in OE-*McWRKY43* under low temperature, and the transcriptional expression trend was consistent with the change of *McWRKY43* during cold stress (Figure 10D), indicating that *McLDOX* did respond to low-temperature regulation in the transient overexpression of *McWRKY43* group in *M. crassipes* leaves.

To sum up, the results showed *McWRKY43* could bind to this fragment, indicating *McWRKY43* may regulate the flavonoids biosynthesis by binding to the W-box in the *McLDOX* promoter to jointly respond to the low-temperature signal, thereby enhancing the low-temperature tolerance of *McWRKY43* overexpressed strains.

## 3. Discussion

### 3.1. McWRKY43 Is a Typical IIc Subgroup Gene

WRKY TFs are classified into different groups, of which Group II WRKY transcription factors can be divided into five subgroups: IIa, IIb, IIc, IId, and IIe. Typical WRKY transcription factors of Group II are one WRKY domain and C-terminal -CX5C23HXH (IIa, IIb, IId, and IIe subgroup) or CX4C23HXH (IIc subgroup). Like homologous sequences such as *AtWRKY43*, the *McWRKY43* sequence motif conforms to the IIc subgroup. Indeed, *McWRKY43* is localized to the nucleus and has transcriptional activation activity in yeast cells (Figure 4A,B). Based on accumulating evidence, group ΙΙc WRKY TFs play complex roles in regulating multiple biological processes in plants [26,27]. Previous studies have reported very diverse functions of group ΙΙc WRKY TFs, such as the active role of the WRKY TF group in regulating flavonoids-mediated *Fusarium oxysporum* resistance in cotton [21], improving drought and salt tolerance [28], regulating heavy metal tolerance [29], and enhancing plant cold tolerance [21]. However, the loss of seed-specific WRKY43 enhanced the tolerance to low temperature during seed germination in *A. Thaliana* [30]. In two kinds of grapes under cold stress, the expression of *VvWRKY43* was upregulated in *V. vinifera* (26-fold), while expression remained low in *V. amurensis* [31]. Sesame *SiWRKY17* and *SiWRKY43* were induced to highly expressed under waterlogging stress, while their homologous genes, *AtWRKY32* and *AtWRKY29*, were responsive to UV irradiation and heat stress, respectively [32]. Some studies indicate that gene expression is also related to hormone homeostasis, phenylpropanoid biosynthesis, sucrose metabolism, and so on [33]. A study also suggests that the orthologous WRKY genes in different species might mediate different pathways and play different roles under abiotic stress response [32]. In our study, low temperature strongly promoted the expression of *McWRKY43*, which increased by about 180 times at 4 h (Figure 3B). Therefore, we speculated that *McWRKY43*, as an IIc subgroup member, may be involved in regulating plant resistance to cold stress.

### 3.2. McWRKY43 Enhances Tolerance to Cold

Cold stress adversely affects plant growth and development and greatly limits the ornamental value of garden plants. In the event of cold stress, there will be a large amount of reactive oxygen species accumulation, so that the mechanical damage caused by the change of the cytoplasmic membranes and the loss of water can lead to cell or plant death [34,35]. So, many biochemical activities will occur to improve the cold damages, such as the enhancement of various osmolytes, antioxidant enzyme activity, and so on [36]. 

Maintaining the integrity and stability of the cell membrane under low-temperature stress is essential for plant cold resistance [34]. Under cold stress, membrane lipid peroxidation affects the structure and function of plant cell membranes, thereby increasing membrane permeability and ROS accumulation. This commenced with structural damage to the cell membrane, i.e., water loss and osmotic potential changes, such as changes in relative electronic conductivity and MDA content. Relative conductivity reflects the degree of cell membrane damage; MDA content can reflect the degree of membrane lipid peroxidation and cell damage [37,38]. In our study, the relative electronic conductivity and MDA content accumulation in the overexpression lines under cold stress were significantly lower than Control (Figure 8A,B). The lipid peroxidation level, assessed by the content of MDA, and its changes are considered to be a standard for the presence or absence of a stress response in them to the influence of various exogenous factors [39]. The relative electronic conductivity and MDA content of the transient transformed leaves in *M. crassipes* were also significantly lower than the Control group under cold stress (Figure S3A,B). These changes indicated that the overexpression of the *McWRKY43* gene reduced the degree of lipid peroxidation at low temperature, maintained cell membrane integrity, and then increased the cold tolerance of *M. crassipes*.

At the same time, plants can also reduce cell osmotic potential by increasing osmotic solute (proline content) under cold stress, so as to maintain cell membrane stability [40]. Plants also undergo oxidative stress responses when exposed to cold adversity. This led to the rapid elevation of reactive oxygen species (ROS), which damaged plant cells [40]. At this time, the antioxidant enzyme system maintains the normal life activities of the plant by effectively clearing excess ROS triggered under environmental stress [41]. Our data revealed significantly higher levels of osmotic solute (proline) content and antioxidant enzymes (POD, SOD) activities in transgenic tobacco subjected to cold stress than in the WT; especially, the activity of SOD was increased by 200% in the OE lines. SOD transformed excessive superoxide radicals into harmless hydrogen peroxide and superoxide anions, while POD quickly broke down hydrogen peroxide into water, neutralizing its damaging effects [37]. In our study, after 4 ℃ cold stress treatment, the WT leaves showed significant wilting and drooping, whereas the transgenic tobacco remained normal in its development (Figure S1). These findings suggest that overexpression of *McWRKY43* regulates reactive oxygen species homeostasis and reduces oxidative damage in tobacco by the accumulation of proline content and antioxidant enzymes activities, and thereby improving cold tolerance.

### 3.3. McWRKY43 Enhances Tolerance to Cold Depending on the Flavonoid Biosynthesis Pathway

Flavonoid compounds are one of the main classes of plant secondary metabolites, which are widely present in plants and play an important role in providing protection against various environmental stresses of plants. Flavonoids act as antioxidants in plants while regulating the antioxidant oxidase system to work together to remove reactive oxygen species. Flavonoids have been reported to play protective roles against abiotic stresses through ROS detoxification, including salt, drought, and cold stress [42,43]. Under extreme environments, the production of antioxidants in plants are limited, resulting in an increase in the intracellular ROS content. In this case, the antioxidant properties of flavonoids help the plant counterbalance the excessive ROS production and repair the damage [43,44,45]. Flavonoids play an important role in coping with cold stress by improving plant physiological characteristics. According to previous research, Pawlikowska-Pawlega et al. [46] approved a previously proposed role for flavonoids in freezing tolerance which flavonoids take part in the protection of cell membranes and proteins against cold stress. Moreover, the increase of anthocyanins in epidermal cells can reduce the osmotic potential of cells and delay freezing through the surface nucleate [47]. In a study on *Liriope spicata*, it was revealed that genes and metabolites involved in the flavonoid pathway had a synergist role in osmoregulation under cold stress [48]. In addition, low temperature upregulated the expression of flavonoid biosynthesis genes such as FLS, increased the flavonoid content in plant tissues [49], and enhanced the cold tolerance of spruce [50]. The expression of anthocyanidin synthase (*BrANS*) genes was sturdily related to cold stress tolerance [51], whereas knockout mutation of *PRODUCTION OF ANTHOCYANIN PIGMENT 1* (PAP1) MYB transcription factor depicts impaired leaf-freezing tolerance in *Arabidopsis* [49]. Many genes associated with the flavonoid biosynthesis pathway are upregulated when suffering from cold stress, including structure genes (PAL, CHI, CHS, C4H, ANS, UFGT, F3H, and DFR) and TFs (MYB, bZIP, WRKY, and bHLH), further confirming that flavonoids play a key role in enhancing cold resistance [52,53,54].

In this study, the flavonoid content in transgenic tobacco and *M. crassipes* leaves was significantly accumulated under low-temperature stress, and the expression levels of flavonoid biosynthesis-related genes such as PAL, CHS, CHI, F3H, DFR, LDOX, ANS, and UFGT were significantly increased (Figure 9 and Figure S4). These results indicated that *McWRKY43* had a positive effect on clearing ROS accumulation during cold stress by promoting the accumulation of flavonoids and being involved in regulating the expression of structural genes in the flavonoid biosynthesis pathway. In particular, the expression levels of *McPAL2* and *McLDOX* were continuously significantly higher than the Control group during low-temperature treatment (Figure S4). In general, WRKY TF is expected to function as a key regulatory protein through precise binding to the W-box (TTGAC (C/T)) that regulates gene expression [55]. In this paper, there are both cis-acting elements in response to low temperature and W-box elements in the *McLDOX* promoter. Through the Y1H assay, it was found that *McWRKY43* could indeed bind to the *McLDOX* promoter. At the same time, qPCR showed that with the time of cold stress, transient expression of *McWRKY43* also enhanced the transcriptional expression of the *McLDOX* gene in *M. crassipes* leaves, and the growth trend of both was consistent. These results suggest that *McWRKY43* confers cold stress tolerance in *M. crassipes* via regulation of flavonoid biosynthesis by binding to the *McLDOX* promoter. Therefore, our future efforts will be focused on further confirming the interacting protein and its role in regulating the expression and function under cold stress. Moreover, it is necessary to study the stable genetic transformation system of *M. crassipes* in order to provide the basis for its genetic breeding.

## 4. Materials and Methods

### 4.1. Plant Materials and Growth Conditions

Two-year-old *Michelia crassipes* plants was used for gene cloning, expression analysis, and transient expression, which was cultivated in potting soil (loam:peat:perlite, 1:1:1). In addition, the seeds harvested by genetic transformation of tobacco were T0 generation, and the positive lines of T0 seedlings were selected as T1 generation for experiments after sowing in the same pot of nutrient-containing soil. *Nicotiana benthamiana*, used for observation of the fluorescence signal, was cultivated in the soil mix. All the plant materials mentioned above were grown in a manual climatic box (RDN-560C-4, Ningbo, China) under day/night conditions of 16 h/8 h (15,000 lx), 24 °C, and 70% relative humidity.

### 4.2. Bioinformatic Analysis of McWRKY43

The protein molecular weight, aa composition, pI, stability, and hydropathicity were estimated by the ProtParam software (http://web.expasy.org/protparam/, accessed on 20 July 2024). The transmembrane domain was analyzed by the TMHMM server 2.0 software (https://services.healthtech.dtu.dk/services/TMHMM-2.0/, accessed on 20 July 2024). The signal peptide was estimated via the SignalP 5.0 Server (https://services.healthtech.dtu.dk/services/SignalP-5.0/, accessed on 20 July 2024). The protein sequences in different species used for multiple sequence alignment analysis were downloaded from the NCBI website (https://www.ncbi.nlm.nih.gov/, accessed on 20 July 2024). The homologous *McWRKY43* sequence alignment with other species was done by DNAMAN 6.0.3, and the phylogenetic tree was generated by NJ of MEGA5.

### 4.3. Expression Pattern Analysis of McWRKY43 M. crassipes Exposed to Cold Stress

Two-year-old *M. crassipes* plants were employed for the cold stress-stimulated *McWRKY43* expression pattern analysis. The top leaves were collected after exposure at 0 °C for 0, 0.5, 2, 4, 8, 24, and 48 h. Total *M. crassipes* RNA isolation was done with a Steady Pure Plant RNA Extraction kit (Accurate Biology, Changsha, China). cDNA was obtained by reverse transcription according to the Evo M-MLV Mix kit (Accurate Biology, China). *McWRKY43* levels were evaluated by qRT-PCR using the LightCycler^®^ 96 Instrument (Roche Diagnostics, Indianapolis, IN, USA). All values were computed via the 2^−∆∆Ct^ relative threshold cycle (Ct) method [56].

Different plant tissues (roots, stems, leaves, and perianth of different colors) were collected for tissue-specific analysis of the *McWRKY43* gene. The expression of *McWRKY43* in different tissues was analyzed by qRT-PCR with *McActin* as the internal reference gene. Primer sequences are summarized in Table S1.

### 4.4. Subcellular Localization

By using the OK Clon DNA Ligation Kit (Accurate Biology, China), full-length *McWRKY43* was inserted into vector p35S: YFP at ECoRI and BamHI sites. The p35S: *McWRKY43*-YFP plasmid and p35S: YFP empty vector were transformed into *Agrobacterium tumefaciens* GV3101 and infiltrated into the *N. benthamiana* leaves, respectively. After infiltration, they were grown in an artificial climate chamber (23/18 °C, 16 h light/8 h dark) for 48 h, and fluorescence signals were detected under the laser scanning confocal microscope (Zeiss LSM710, Jena, Germany) [42]. All primers are shown in Table S2.

### 4.5. Transactivation Activation Analysis in Yeast

For transcriptional activation activity assays, the full-length CDS of *McWRKY43* was constructed into the yeast pGBKT7 vector and then introduced into yeast strain AH109 together with pGADT7 following the yeast protocol manual (WeidiBio, Shanghai, China). pGBKT7+ pGADT7 were used as negative Controls. The transformants were spotted onto the yeast synthetic dropout medium (SD/-Leu/-Trp and SD/-Leu/-Trp/-His/-Ade) (Coolaber, Beijing, China) and incubated at 29 °C for 3 days before observation. The transcriptional activation activities were evaluated according to yeast growth status. A total of 20 g/L agar and carbon sources were added to the medium according to the SD Broth protocol manual.

### 4.6. McWRKY43 Overexpression in Transgenic Tobacco

The p35S-*McWRKY43* plasmid was incorporated into the *Agrobacterium tumefaciens* strain EHA105 using the freeze–thaw procedure. In turn, the strains were transferred to tobacco (*Nicotiana tabacum*) by the leaf disc transformation. The transgenic tobaccos were verified by PCR and qRT-PCR. The primers used are summarized in Table S3.

### 4.7. Cold Treatment in Transgenic Tobacco Seeds

Wild-type tobacco seeds and transgenic tobacco seeds overexpressing *McWRKY43* were sown on 1/2 Murashige and Skoog (MS) medium and vernalized at 4 °C for 2 days. Subsequently, the seeds were grown in a manual climatic box in a 10 °C [56] (16-h light/8-h dark), and seed germination was recorded each day. Three independent experiments were performed, with 70 seeds per experiment.

### 4.8. Phenotypic Assay and Measurement of Physiological Indicators

All tobacco plants were cultivated in an artificial climate chamber (relative humidity 70%, 22/18 °C, 16 h light/8 h dark). After 2 months of culture, the wild-type and three transgenic tobacco lines with normal growth were divided into two groups. In the first group, all the tobacco was still growing normally in the artificial climate chamber at 22 °C. The second group was exposed to cold stress at 4 °C for 24 h [40]. Samples of wild-type and transgenic tobacco leaves before and after low temperature were used to determine physiological indexes. The flavonoid content and antioxidant enzyme activities, namely SOD and POD, were assessed with reagent kits (Suzhou Corning Biotechnology Co., Ltd., Suzhou, China). Finally, a conductivity meter (Mettler Toledo, FiveEasy Plus FE38, Shanghai, China) was employed for the measurement of relative electronic conductivity.

### 4.9. Transient Expression in Michelia crassipes Leaves

Recombinant plasmid of the *McWRKY43* target gene was introduced into Agrobacterium EHA105. A total of 500 μL bacterial solution was transferred to LB liquid medium and cultured for 18h in a shaker at 28 °C, 200 rpm, centrifuged at 4000× *g* for 10 min and then re-suspended in an equal volume infiltrating solution (10 mM MgCl_2_, 10 mM MES pH 5.6 + 200 μM acetylsyringone). Finally, the resuspended cells were then diluted to OD600 = 0.8 with infiltration solution. After static culture for 3 h, the Agrobacterium solution was ready for infiltration. The annual leaves of 2-year-old *M. crassipes* was transformed by injection of Agrobacterium strain cells harboring corresponding plasmids (agroinfiltration solution (CK) and *McWRKY43* expression vectors) to the abaxial surface using a 1 mL disposable needleless syringe, respectively. After injection, plants grew in darkness for 1 day. Then, the treated plants resumed their growth under 16 h light/8 h dark until the samples were harvested for gene-expression pattern analysis and cold treatment [57].

### 4.10. Quantitative Real-Time PCR

Two-month-old wild-type and transgenic tobacco were sampled under 4 °C cold stresses for 12 h. With reference to Section 4.3, the expression patterns of structural genes in flavonoid biosynthesis pathway under cold stress were analyzed using *NtEFIα* as the internal reference gene.

According to Section 4.9, *McWRKY43*, instantaneous transformation was carried out in the leaves of *M. crassipes*. The expression level of *McWRKY43* was analyzed by sampling 24 h after injection to determine the feasibility of instantaneous transformation. Then, leaves of 2-year-old wild-type and instantaneous *McWRKY43* were collected after being treated at 0 °C for 0, 0.5, 2, 4, 8, 12, and 24 h, respectively. The expression level of structural genes in the flavonoid biosynthesis pathway was evaluated by qRT-PCR. Primer sequences are summarized in Table S4.

### 4.11. Yeast One-Hybrid Assay

The specific primers were designed according to the sequence of yeast single-hybrid vector pGADT7 and *McWRKY43* gene (Table S5). Using the OK Clon DNA Ligation Kit (Accurate Biology, China), full-length *McWRKY43* was inserted into vector pGADT7 at the NdelI and ECoRI sites to obtain recombinant vector PGADT7-*McWRKY43*. According to the results of *McLDOX* promoter analysis, three tandem copies of W-box cis-acting elements were cloned into pHIS2 vector as a reporter vector, using primers as shown in supplementary Table S4. After that, the pHIS2-Pro-*McLDOX* vector was transformed into yeast strain Y187 (WeidiBio, Shanghai, China) together with pGADT7-*McWRKY43*, and were plated on DDO(SD/-Leu/-Trp) and TDO (SD/-Leu/-Trp/-His) media supplemented with 50 mM 3-AT. pGADT7-53+p53HIS2 was used as positive Control. The growth of yeast was observed after 3 days at 29 °C [58].

### 4.12. Statistical Analysis

SPSS 19.0 software was used to analyze the statistical significance and plotted with Origin 2021. All data were the mean ± standard deviation (SD) of three biological replicates, with three technical replicates per biological replicate. Statistical significance was determined by one-way ANOVA. Multiple comparisons were tested by Tukey’s test, and significant differences (*p* < 0.05) were indicated by different lowercase letters.

## 5. Conclusions

*MCWRKY43*, a WRKY TF, was extracted from *M. crassipes*, and its protein was located in the nucleus. The *McWRKY43* expression was positively correlated with cold stress in *M. crassipes*. Based on our analysis, *McWRKY43* is an essential gene that markedly enhances flavonoid accumulation, antioxidant enzymes activities, and maintains membrane permeability and eventually confers cold stress in tobacco. *McWRKY43* may be involved in the regulation of flavonoid biosynthesis pathway in combination with *McLDOX* promoter and upregulates the expression of related structural genes to enhance the resistance to cold in *M. crassipes*. Our conclusion would provide a theoretical basis for future studies attempting to enhance cold stress tolerance by flavonoid in *M. crassipes*.

## Data Availability

The data presented in this study are available on request from the corresponding author.

## Supplementary Materials

The following supporting information can be downloaded at: https://www.mdpi.com/article/10.3390/ijms25189843/s1.
