# Supplementary material for: McWRKY43 Confers Cold Stress Tolerance in Michelia crassipes via Regulation of Flavonoid Biosynthesis
Source: Int J Mol Sci. 2024 Sep 12;25(18):9843. doi: 10.3390/ijms25189843 (PMC11432407; doi:10.3390/ijms25189843)
Supplement: Supplementary file 1 [file ijms-25-09843-s001.zip › ijms-3148478-supplementary.pdf]

Table S1. Primer sequences of expression pattern analysis of *McWRKY43*

Primer name	Primer sequence
McWRKY43-F	AATTCGTAATCATGTCATAGCTGT
McWRKY43-R	CACCCCAGGCTTTACACTTT
McActin-F	ATGGGGTATTTGAGGGTGAGG
McActin-R	AGGCTGGATTTGCTGGTGAT

Table S2. The Primers of Subcellular Localization ad Transactivation Activation Analysis in Yeast

Primer name	Primer sequence
McWRKY43-YFP-F	atgggatctactagtgaattcATGCAAGGAGACGCACCGA
McWRKY43-YFP-R	gggggtaccgtcgacggatccAAATCTTGAAAGATATTGCATCTGCT
pGBKT7-McWRKY43-F	tcagaggaggacctgcatatgATGCAAGGAGACGCACCGA
pGBKT7-McWRKY43-R	tcgacggatccccgggaattcTTAAAATCTTGAAAGATATTGCATCTGC

Table S3. The Primers for the identification of transgenic tobacco plants

Primer name	Primer sequence
OE-McWRKY43-F	TCTCTGTCGACTCTAGAGGATCCATGCAAGGAGACGCACCG
OE-McWRKY43-R	AACGATCGGGGAAATTCGAGCTCTTAAAATCTTGAAAGATA
NtEF1α-F	TGGTTGTGACTTTTGGTCCCA
NtEF1α-R	ACAAACCCACGCTTGAGATCC

Table S4. Structural genes related to flavonoid biosynthesis pathway in tobacco and *M. crassipes*

Primer name	Primer sequence
NtCHI-F	GTCAGGCCATTGAAAAGCTC
NtCHI-R	CTAATCGTCAATGCCCCAAC
NtCHS-F	TTGTTCGAGCTTGTCTCTGC
NtCHS-R	AGCCCAGGAACATCTTTGAG
NtF3H-F	CAAGGCATGTGTGGATATGG
NtF3H-R	TGTGTCGTTTCAGTCCAAGG
NtDFR-F	AACCAACAGTCAGGGGAATG
NtDFR-R	TTGGACATCGACAGTTCCAG
NtANS-F	TGGCGTTGAAGCTCATACTG
NtANS-R	GGAATTAGGCACACACTTTGC
NtUFGT-F	GAGTGCATTGGATGCCTTTT
NtUFGT-R	CCAGCTCCATTAGGTCCTTG
Mc*PAL1*-F	GAATGCCGGTCATACCCGCTGTA
McPAL1-R	GAGCCATCCCATCCATCCAAA
McPAL2-F	GTGGCTGAGTGCCGGTCTTAC
McPAL2-R	CGATCTGTTCACCAGGCGACA
McPAL3-F	GGGGAGGTGATGATGAAGTGC
McPAL3-R	ACCTTATCAAACCACCCCCAGTA
McPAL4-F	GGACCATGCGCTGATTAACGG
McPAL4-R	TTCGGCAAATGGGTCTTGAGC
McPAL5-F	AGTCGAATCCACCCGAAATGC
McPAL5-R	CCCGTCGCTGATCGCAACAAA
McCHS1-F	CGGTTGGTCTATACAAGCCATTA
McCHS1-R	CAACAACGGGAAGGTTGGAGT
McC4H-F	GTGAGAAAGGCGGGCAGTTCA
McC4H-R	CACCGTTCCATCCTCTTACCAAC
McF3'H-F	CACCTTCTGCGCCGAATATGGAC
McF3'H-R	GGAAAAGGATCTCGCGGATGACT
McDFR-F	TTCGTCAGTGGACCACGCTAG
McDFR-R	AGCTCTGGCGGGCTTCCAT
McLDOX-F	CGACGGAATCCAACGAAGGT
McLDOX-R	ACGGTCGATATGGAATCCTTGC

Table S5. The primers of Yeast One-Hybrid Assay

Primer name	Primer sequence
PGADT7-*McWRKY43*-F	gtaccagattacgctcatatgATGCAAGGAGACGCACCGA
PGADT7-*McWRKY43*-R	atgcccacccgggtggaattcTTAAAATCTTGAAAGATATTGCATCTGC
pHIS2-Pro-*McLDOX*-F	AATTCTTGACCTTGACCTTGACCTTGACCTTGACCGAGCT
pHIS2-Pro-*McLDOX*-R	CGGTCAAGGTCAAGGTCAAGGTCAAGGTCAAG

Table S6. The germination rate of WT and transgenic tobacco at 10 °C

	12d	17d	22d	27d	32d	37d	42d	47d	52d	57d
WT	0.0%±0.00 b	1.4%±0.01 b	2.9%±0.07 b	7.1%±0.01 b	14.3%±0.14 b	22.9%±0.71 b	35.7%±0.00 b	52.9%±0.71 b	60.0%±0.71 b	61.4%±0.71 b
OE 1	1.4%±0.08 a	5.7%±0.06 a	10.0%±0.01 a	20.0%±0.01 a	30.0%±0.14 a	41.4%±2.14 a	65.7%±0.00 a	81.4%±0.71 a	88.6%±0.71 a	90.0%±2.14 a
OE 2	2.9%±0.42 a	7.1%±0.00 a	10.0%±0.01 a	22.9%±0.07 a	31.4%±0.14 a	42.9%±1.42 a	67.1%±0.71 a	88.6%±1.43 a	92.9%±1.42 a	92.9%±0.71 a
OE 3	1.4%±0.08 a	7.1%±0.07 a	10.0%±0.07 a	18.6%±0.14 a	27.1%±0.14 a	44.3%±1.42 a	62.9%±1.42 a	74.3%±4.28 a	84.3%± 2.14 a	87.1%±2.14 a

Note: Values represent means ±SD of three replicates with 70 seeds per replicate, and different lowercase letters indicate significant differences by Tukey's test ($p < 0.05$).

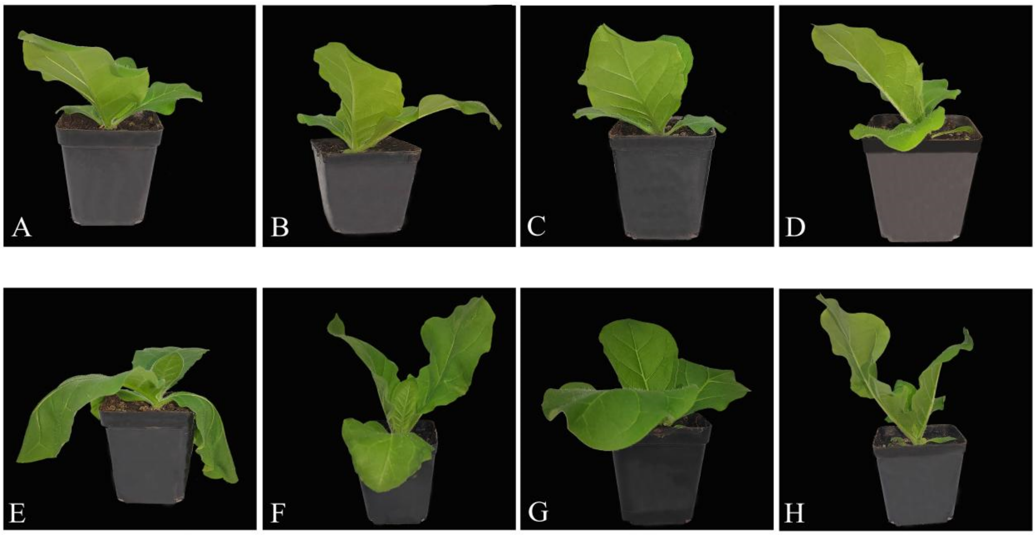

Figure S1. Overexpression of *McWRKY43* in tobacco enhances plant tolerance to cold stress. Performance of WT and *McWRKY43* transgenic plants after low temperature treatments. (A-D) Wild type and three overexpressed *McWRKY43* transgenic lines in normal environment; (E-H) Wild type and three overexpressed *McWRKY43* transgenic lines under cold stress.

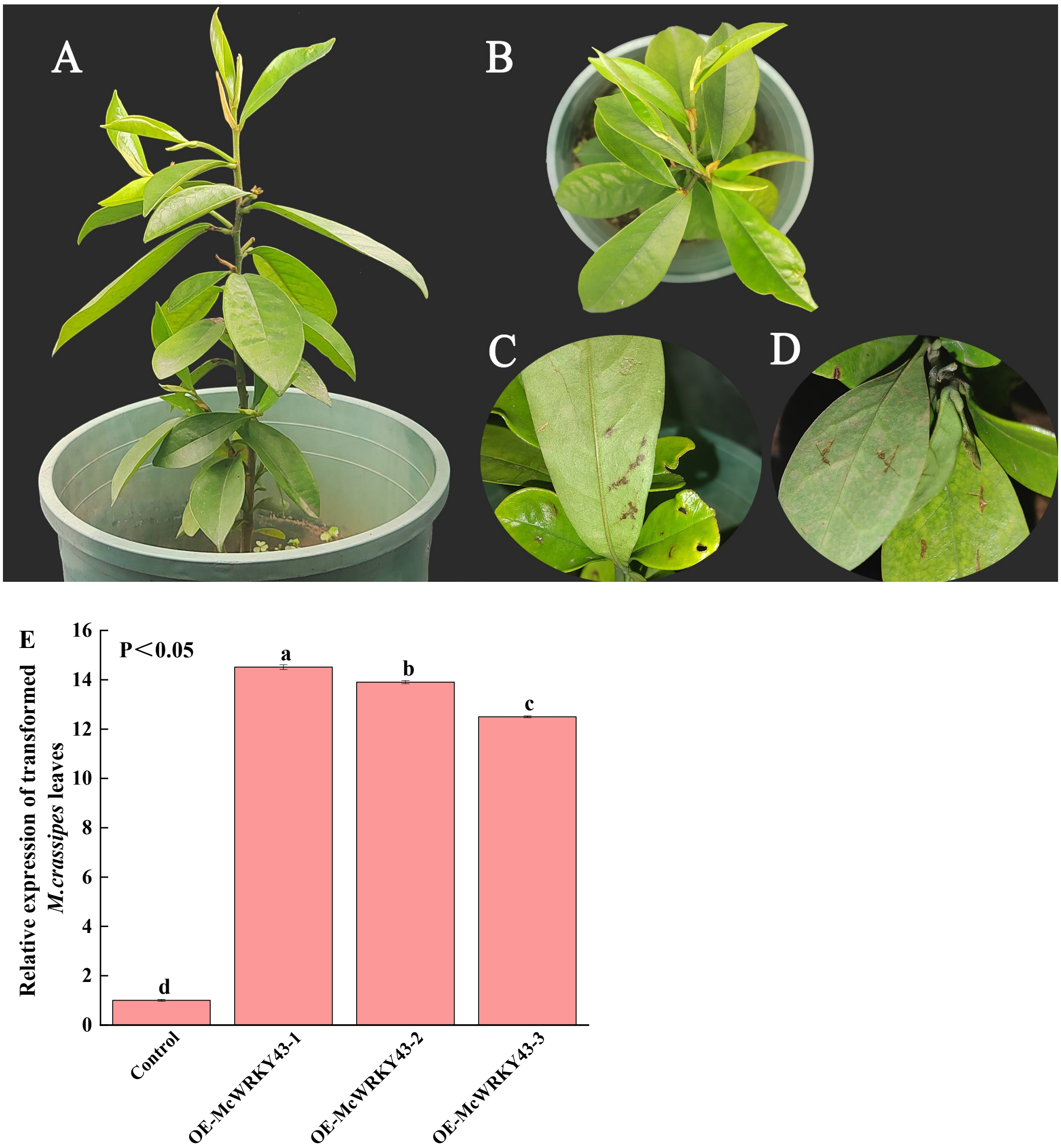

Figure S2. Transient over-expression of *McWRKY43 M.Crassipes* leaves were infiltrated with cDNA constructs corresponding to *McWRKY43* and agroinfiltration solution (CK). (A-B) Annual *M.Crassipes* seedling-plant. Front view(A) and top view (B). (C-D) The phenotypes of leaves of *M.Crassipes* after being infiltrated. Transient overexpression of *McWRKY43* (C) and CK (D). (E) *McWRKY43* gene relative expression level of transformed *M.Crassipes* leaves after being infiltrated 2 days. The values represent the mean ± SD of three replicates, and different letters above the bars as determined by Tukey's test indicate significant differences at the $p < 0.05$ level.

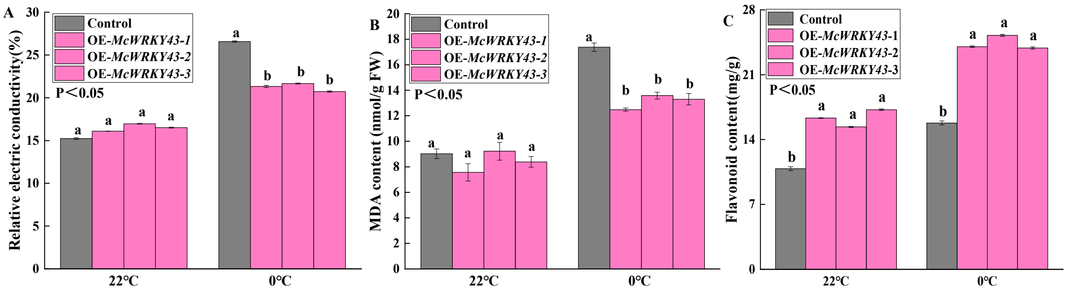

Figure S3. Measurement of Membrane permeability indices and flavonoid content in *M.Crassipes* leaves. (A-C) Relative electronic conductivity, MDA content and flavonoid content. The values represent the mean ± SD of three biological replicates, and different letters above the bars as determined by Tukey's test indicate significant differences at the $p < 0.05$ level.

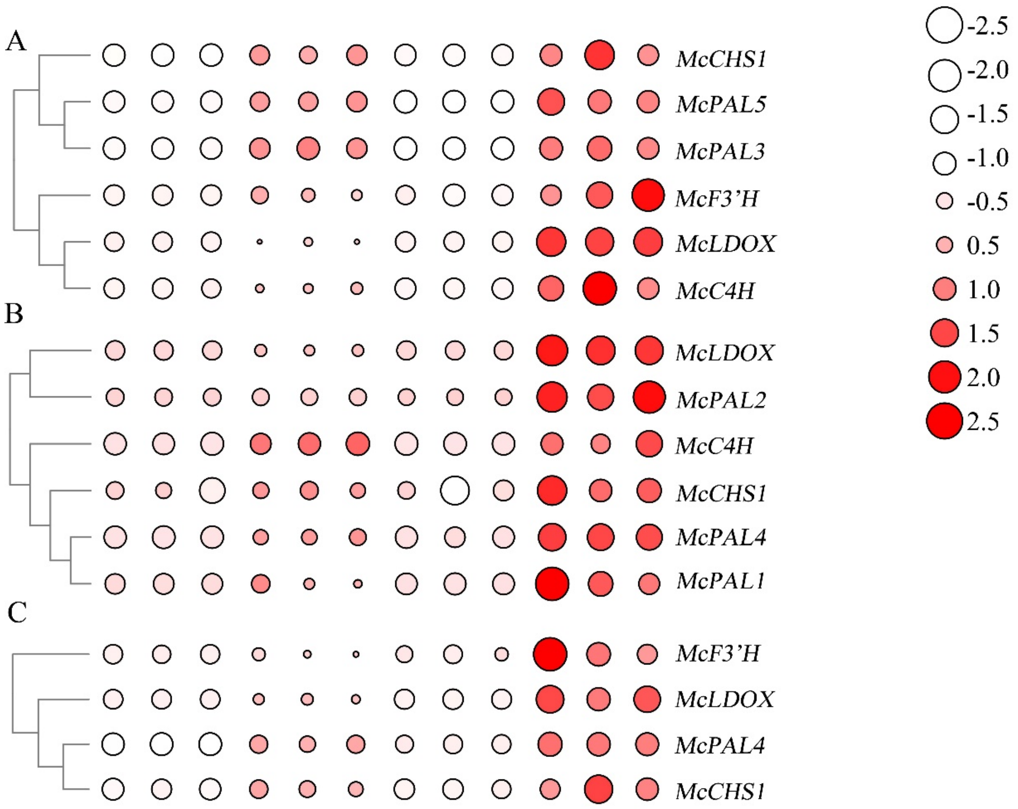

Figure S4. Heatmap of flavonoid biosynthesis-related genes in Control and OE-*McWRKY43* in *M.Crassipes* leaves under cold stress. (A-C) The expression levels of related flavonoid biosynthesis-related differential genes were respectively 0.5 h (A), 4 h (B) and 24 h (C) under cold stress.

>McLDOX

GTGTACGACTCAAAAGTTTGTAGACTAGGCACGTAGGTAACTCAAATTAAACTCTCCA
ACTCACCAAACCCAACTTAGAAAATAAACCTAAACTAATACTAGTTGGCTGATTGAAA
CCTTCCATTTATACACATTTGATTACAACTAGTTTTACGTCCAACGTCCATTAATTACCAG
CAAATCAACTAGTTAACACATTACTTTCTGTTCAAACGTTGTTATATAAACCATCTAAAA
TAGGGCCCACTATTTAAACGGTTTAGGTTTAGGTATTATATATAAGCTACATGTGAAATTT
TTGAGTGCCTGTGCGTCATCTATAGCACTCTGGCAGAAAAAAAAAATAAATGCAAATA
CTAAATCAACAAAGCGTGGAAGTTTGATCACATTGTTAGACAGAAGATGAACCGTCTG
GATGATCCACTAACATTGGGCCATATGTATTTTCATGGGCTGCAACGAACAAACGCCAG
TATATCGAGACGGAAGAGTAATCTTCTTAAAACTCACAAATAGCACGTTCTTACTTGTG
TCAACTTCACAGCGAAAGGAAAATGGACTTTGGTTTCTTAGAAGTCGCCAACAACAAC
CCATCTTTTTCTTCTTTTTTGCTTCGGTGGGACATCTTTGAAAGTTCCCCACCATTTCTT
AAGTTGTGGCGTCTCATTCACCGTACACGCGGCATGCATGCACGTCAAAACAGGCTGT
CTAGATGTGGGCCCACTGTGGATGGAACAATACCAAAAAAGGAACTCATGGATTGATA
ATCCTAACCATCCAATCGATATGCGTATCAATTGGACGTTATCAGTGGTTCCTGGGATGT
AGCTCGGACAGGTAGGGTTAGGTTGAGGGTTAATTCGGGCCCAACCAGACCTTGAGA
GTTAAGTTCCTGCGCTGGGAACCCAGGTGGGGCCACTGCGATGTTTATGAGAATTCCA
AACCGTTCATCCGTTTTTTGAGTTGATATTAGGATATGAGAACAAAGATAAACCGGATC
CAATGCTTAAGTGGGCTGAAGACATGAGAATTGAACGTCCAAAGTCGAAATATTCGTG
GGGCCGCATAAGTTTTGAATCATGCTAATATTTGTGTCTTCAGTTAATCCCAGTAGGGAT
GAGGTTATGAACAGTATAGATGGCATGTAAACATCACTATCTACCACAGGGTGGTTTCA
ACGGTAGAATTTTCCTAACCACTTTCTCTTTTAGTACGGCCTACTGGACTCATGGATCCT
GCTTACTTTGGTTTCATAA**CCGAAA**ATGAGCACAAAAAAACGGATGGACGGAGTGGATT
LTR
CCTCACAAACATCACGGTGGCCCCACCTCAGTTTCCACCGCACGAACTTCATGAGAAA
GGCTTACCCCAAATTCCAAACTGATGTTTCCAACCTTAATCCTAACTGAATTCGTCTCTA
CTCAACCCTAGACCAACCCAACCCGACCTAACCGGAACCTAGTACATTTGATTACAAC
CAAGCCGGCTTGAATTTGCTTAAACAGAACCTACCCTTATTGGGTTGAAGTCTAATTTC
GAACCCTAATCCAATTTTTGGATCTTGACAAACCTGAACCAACCTGACCCAGGACCTG
GATTTGGGTTGGGAGCAATGGGACCCAATTAGTTGCAGTCCTATTGATCCCAACTCATG
GAGCGAAAATCACATGGTAAGATGGT**TTGACC**AGTGTGAACTTGAGACTGAGCTCTAT
W-box
CAGCACGCACTGCCTACTTTCTGCACATTCCAAAATGTTTTAAATGTATGACATGTGCG
CAGGTGGGACAAGAGACCACAACATAAAAAGTTGTCTTTGCCCATCCATTATTAGACG
ATTAAACCTCTCTTCTATTTATATGCATGGGTTCTAACCGTTGTCTATCATCGTACACTAC
AATATTTATATTTTTTTGTGTGTGTTCCTGTTCGAAAACCAACGGTCGATATG

Figure S5. *McLDOX* promoter sequence
